# An Evaluation of the Effect of Social Media Platforms on the General Population's Decision-Making About Cosmetic Procedures in Makkah City, Saudi Arabia

**DOI:** 10.7759/cureus.41093

**Published:** 2023-06-28

**Authors:** Eyad E Sindi, Mohammed F Bondagji, Jihad A Malibary, Mohammed K Alghamdi, Doaa S Baashar, Samaa A Sindi, Abdulrahman M Almalki, Faris Alsaedi, Homaid O Al-Otaibi

**Affiliations:** 1 Department of Medicine and Surgery, Faculty of Medicine, Umm Al-Qura University, Makkah, SAU; 2 Department of Dermatology, King Faisal Hospital, Makkah, SAU; 3 Department of Dermatology and Laser Dermatosurgery, King Faisal Hospital, Makkah, SAU

**Keywords:** cosmetology, mobile phone, social media, cosmetic, aesthetic

## Abstract

Background and objective

Aesthetic procedures are one of the most commonly performed medical procedures. Surgical and non-surgical cosmetic treatments that are routinely performed include breast augmentations, rhinoplasty, botulinum toxin, and fillers. Several factors contribute to the increase in the popularity of these procedures, including body image dissatisfaction, the opinion of peers, and surgeon-related factors such as the surgeon's reputation, board certification, or years of experience. In addition, recent evidence suggests that active and passive usage of highly visual social media that focus on appearance-centric content have been positively associated with the acceptance of, and desire for, cosmetic procedures. In this study, we aimed to investigate the influence of social media on individuals' decision-making in terms of cosmetic procedures.

Method

The setting for this population-based cross-sectional study was public places in Makkah City, Saudi Arabia, and it was conducted from September to November 2021. Our study included adults above the age of 18 years. The exclusion criteria were as follows: non-Arabic speakers and individuals with congenital anomalies or dysmorphic dermatological diseases. The final sample consisted of 364 participants. All analyses were carried out using IBM SPSS Statistics version 28 (IBM Corp., Armonk, NY). Continuous and categorical variables were compared using ANOVA and Pearson's Chi-squared test.

Result

A total of 364 participants were included in the study, and 80% of them used their phones on a daily basis for >4 hours per week. The mean age of the participants was 27.4 ± 8.3 years, and they had a mean BMI of 25.0 ± 6.4 kg/m^2^; 60% of the participants were female. Participants with a history of cosmetic procedures or a desire to undergo cosmetic procedures reported a similar pattern of daily phone use. There was no significant association between the reported history of cosmetic procedures and the daily duration of selected social media platform use.

Conclusion

We found no significant correlation between the hours spent on social media and the participants' history or desire for cosmetic procedures. Only 54 subjects compared themselves to social media celebrities. These findings could be attributed to the fact that the Makkah population is conservative in their religious attitudes and traditions, which makes them less susceptible to social media influences.

## Introduction

The popularity of aesthetic procedures has been on the rise in the past few years and these are some of the most frequently performed medical procedures [1]. Surgical and non-surgical cosmetic treatments include breast augmentations, rhinoplasty, botulinum toxin, and fillers. Cosmetic procedures range from reconstructive surgery to those treating pathological conditions [2]. Multiple factors contribute to the increase in the popularity of these procedures. Poor body image is one of the strongest predictors of a patient's interest in cosmetic procedures. Social media also plays a role, as do surgeon-related factors such as the surgeon's reputation, board certification, or years of experience. Films, magazines, and other media have also contributed to people's desire to undergo cosmetic procedures [1-3].

Cosmetic procedures have increased in tandem with the continuous evolution of modern society, where the well-being of individuals is also dependent on the attractiveness of their physical appearance according to various societal norms [4]. Although the legality of reconstructive surgery has never been questioned, the limits and legitimacy of aesthetic surgery have long been the subject of discussion and debate. Based on the data from the American Society for Aesthetic Plastic Surgery, the number of cosmetic procedures has increased by 446% since 1997 and by 8% overall in 2007. There was a 17% increase in the number of men having cosmetic surgery [5]. The American Society for Aesthetics reported 2,314,720 cosmetic surgical procedures in 2020. Rhinoplasty is the most common procedure performed [6]. Conversely, the UK has seen a decrease in the number of cosmetic procedures. The number of procedures carried out by the members of the British Association of Plastic Surgeons decreased by 27% between 2020 and 2021 [7]. Another Iranian study reported that issues regarding self-esteem, dissatisfaction with body image, and matters pertaining to conformity influence the acceptance of cosmetic procedures [8]. A recent study shows that social media negatively impacts body satisfaction, especially in adolescents and young adults, i.e., they tend to compare their appearance with celebrities and social media, which often leads to negative perceptions about their own bodies and appearance [9].

In Saudi Arabia, YouTube, Instagram, Facebook, and Twitter are the most popular social media platforms, and these are much more popular than TikTok and Snapchat. Most users fall between the ages of 25 and 34 years [10]. Worldwide data have revealed that people spend two hours and 24 minutes per day on social media on average [11]. In Saudi Arabia, people spend an average of three hours and six minutes [10]. Recent evidence suggests that active and passive usage of highly visual social media applications like Instagram, Snapchat, and TikTok, which deal with appearance-focused content, have been associated positively with the acceptance of, and desire for, cosmetic procedures [12]. In particular, we assume that following social media influencers who undergo cosmetic procedures may significantly impact young adults’ attitudes toward cosmetic procedures [13]. The normalization of cosmetic procedures may also be due to influencers' expression of openness and appreciation for their cosmetic procedures, which generally lowers the threshold for cosmetic procedures [12]. In this study, we explored the influence of social media on individuals' decisions to undergo cosmetic procedures in Makkah City, Saudi Arabia.

## Materials and methods

Study design, population, and sampling

Using a convenience sample technique, this descriptive cross-sectional study was conducted in public places such as malls, gardens, and cafes in Makkah City, Saudi Arabia, between September 2021 and November 2021. Ethical approval was obtained from the Biomedical Ethics Committee of the Faculty of Medicine at Umm Al-Qura University (UQU), Makkah, Saudi Arabia (IRB approval no: HAPO-02-K-012-2021-09-764). The study included adult males and females above the age of 18 years except for non-Arabic speakers and individuals with any congenital anomalies or dysmorphic dermatological diseases. According to the General Authority for Statistics, the population in Makkah City is approximately 8,325,304 individuals. The target sample size for this study was calculated using OpenEpi version 3.0 with a confidence interval (CI) level of 95% and an anticipated frequency of 50%. The sample size was determined to be 385 participants. However, after approaching the individuals, a total of 364 participants were ultimately included in the study.

Data collection and instrument survey

A questionnaire was developed in Arabic so that it was easy to comprehend for the participants. The questionnaire was distributed in a standard way to all study participants. The data were collected using an electronic device (iPad) that contained the questionnaire created by using Google Forms. Participants were asked to provide consent before filling out the questionnaire. The questionnaire was divided into four sections. Section 1 contained the consent form necessary for the study to proceed. Section 2 comprised questions regarding sociodemographic characteristics such as age, gender, and level of education. Section 3 involved questions about the participants' social media usage, including the number of hours spent using the phone in general or social media applications in particular. These data were collected with the help of data collectors who instructed the participants on how to calculate the exact average hours of their weekly usage from the mobile phone settings. The apps surveyed included WhatsApp, Twitter, Snapchat, Instagram, YouTube, and Facebook. Section 4 included questions about prior cosmetic procedures, acceptance of such procedures by their family and friends, and any apprehensions or shame experienced.

Statistical analysis

Continuous and categorical variables were compared using ANOVA and Pearson's Chi-squared test, respectively. A partial correlation that controlled for categorical sociodemographic variables was run between selected social media platforms and topics of interest. All analyses were conducted using IBM SPSS Statistics version 28 (IBM Corp., Armonk, NY).

## Results

We included a total of 364 participants in the study. The mean age of the participants was 27.4 ± 8.3 years and they had a mean BMI of 25.0 ± 6.4 kg/m^2^; 60% of the participants were female. Regarding educational status, more than half of the participants are undergraduates. Most of the participants were Saudis. Of note, 79% of the participants used the phone daily for >4 hours per week. There were no significant differences in the hours spent on the phone across age, BMI, sex, education, nationality, employment, and income (Table 1).

**Table 1:** Sociodemographic variables and average hours of daily phone use in a week SD: standard deviation; df: degrees of freedom

	Phone usage: <2 hours, n (%)		2–4 hours, n (%)		≥4 hours, n (%)		Total, n (%)				
	5 (1%)		72 (20%)		287 (79%)		364 (100%)				
	Mean	SD	Mean	SD	Mean	SD	Mean	SD	df	F	P-value
Age, years	25.4	-4	29.1	-8.8	27	-8.2	27.4	-8.3	2	2.083	0.126
BMI, kg/m^2^	22.4	-3.9	24.5	-4.5	25.2	-6.8	25	-6.4	2	0.779	0.459
	N	%	N	%	N	%	N	%	df	Chi-squared	p-value
Sex									2	0.891	0.64
Female	2	1%	44	20%	174	79%	220	100%			
Male	3	2%	28	19%	113	79%	144	100%			
Education									6	9.458	0.15
High school	3	3%	21	22%	73	75%	97	100%			
Bachelor's degree	0	0%	6	38%	10	63%	16	100%			
Undergraduate	2	1%	36	16%	181	83%	219	100%			
Postgraduate	0	0%	9	28%	23	72%	32	100%			
Nationality									2	4.1	0.13
Saudi	5	1%	62	19%	267	80%	334	100%			
Non-Saudi	0	0%	10	33%	20	67%	30	100%			
Employment											
Employee	1	1%	34	23%	111	76%	146	100%	10	16.936	0.08
Freelancer	1	6%	7	41%	9	53%	17	100%			
Retired	0	0%	1	25%	3	75%	4	100%			
Unemployed	1	4%	5	21%	18	75%	24	100%			
Student	1	1%	21	14%	130	86%	152	100%			
Housewife	1	5%	4	19%	16	76%	21	100%			
Income, Saudi riyal									8	4.305	0.83
<5,000	4	2%	45	19%	186	79%	235	100%			
5,000–9,999	0	0%	10	16%	52	84%	62	100%			
10,000–19,999	1	2%	12	26%	34	72%	47	100%			
20,000–29,000	0	0%	4	31%	9	69%	13	100%			
≥30,000	0	0%	1	14%	6	86%	7	100%			

**Figure 1:**
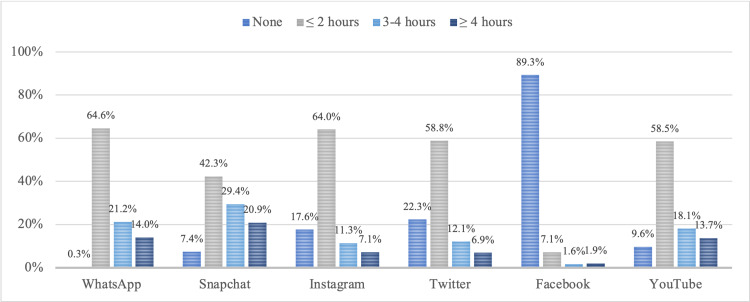
Daily social media usage (average hours in a week)

Figure 1 depicts the number of hours the participants spent on the selected social media platforms daily; more than half of the participants spent more than three hours on Snapchat and less than two hours on WhatsApp, Instagram, Twitter, and Youtube. The least used social platform was Facebook (Figure 1).

**Figure 2:**
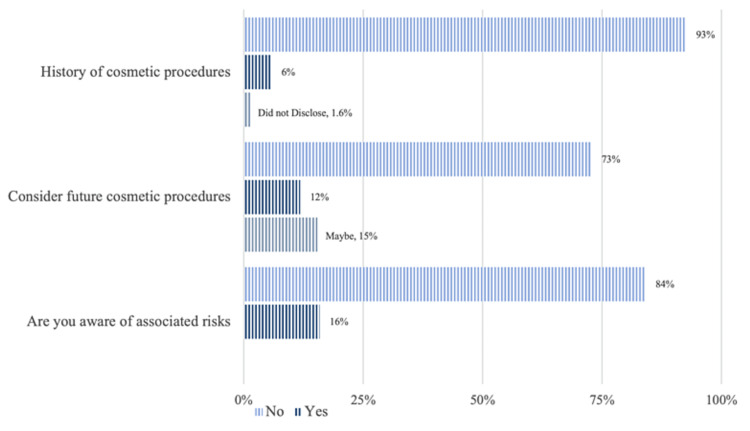
Attitudes toward cosmetic procedures

**Figure 3:**
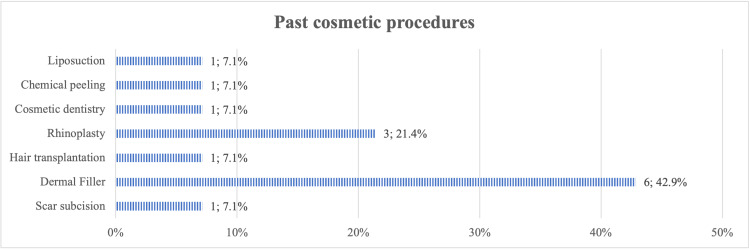
Proportion of participants who underwent different types of cosmetic procedures in the past

**Figure 4:**
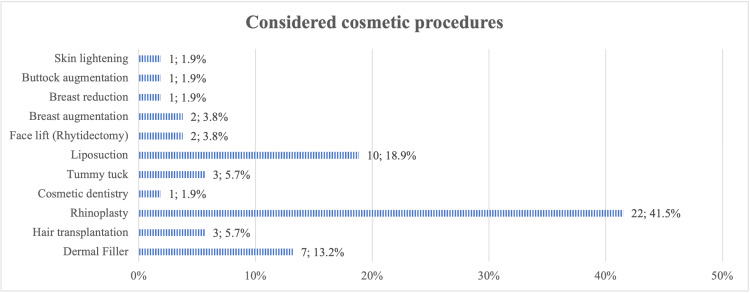
Proportion of participants in terms of the various desired cosmetic procedures that they wish to undergo

Figure 2 illustrates the participants' attitudes toward cosmetic procedures. Only 6% had undergone a cosmetic procedure in the past; 12% said they considered undergoing future cosmetic procedures, while 15% chose maybe when asked if they would consider cosmetic procedures in the future. Most of the participants (84%) were unaware of the risks associated with cosmetic procedures. Dermal fillers were the most common cosmetic procedure that the participants had undergone in the past (43%), followed by rhinoplasty (21%) (Figure 3). In comparison, rhinoplasty was the most desirable procedure that they wished to undergo in the future (42%), followed by liposuction and dermal fillers (19% and 13%, respectively) (Figure 4).

Those who compared themselves to celebrities spent more than four hours daily on mobile phones (91%). Similarly, those who had a past history of cosmetic procedures or those who said they would consider it in the future spent more than four hours daily on mobile phones (86%). However, the association was not statistically significant (p=0.06, 0.65, and 0.55, respectively) (Table 2).

**Table 2:** Attitudes toward cosmetic procedures and hours of daily phone use df: degrees of freedom

	Phone usage: <2 hours		2–4 hours		≥4 hours		Total				
	N	%	N	%	N	%	N	%	df	Chi-squared	P-value
Do you compare yourself to celebrities?									2	5.557	0.06
Yes	0	0%	5	9%	49	91%	54	100%			
No	5	2%	67	22%	238	77%	310	100%			
Do you consider undergoing cosmetic procedures in the future?									4	3.055	0.55
No	5	2%	54	20%	206	78%	265	100%			
Yes	0	0%	6	14%	37	86%	43	100%			
Maybe	0	0%	12	21%	44	79%	56	100%			
Have you done a cosmetic procedure before?									4	2.484	0.65
Did not disclose	0	0%	0	0%	6	100%	6	100%			
Yes	0	0%	3	14%	18	86%	21	100%			
No	5	1%	69	20%	263	78%	337	100%			

There was no statistically significant association between the history of cosmetic procedures and the daily hours spent on each social media platform separately (Table 3). In contrast, participants who spent less time on WhatsApp and Twitter were more likely not to consider future cosmetic procedures (p=0.05 and 0.04, respectively) (Table 4).

**Table 3:** Association between the history of cosmetic procedures and daily hours spent on different social media platforms df: degrees of freedom

	Underwent cosmetic procedures: Yes		No		Did not disclose		Total				
	N	%	N	%	N	%	N	%	df	Chi-squared	P-value
	21	6%	337	93%	6	1%	364	100%			
WhatsApp									6	0.791	0.99
None	0	0%	1	100%	0	0%	1	100%			
<2 hours	14	6%	218	93%	3	1%	235	100%			
2–4 hours	4	5%	71	92%	2	3%	77	100%			
≥4 hours	3	6%	47	92%	1	2%	51	100%			
Snapchat									6	3.995	0.68
None	2	7%	25	93%	0	0%	27	100%			
<2 hours	5	3%	146	95%	3	2%	154	100%			
2–4 hours	9	8%	96	90%	2	2%	107	100%			
≥4 hours	5	7%	70	92%	1	1%	76	100%			
Instagram									6	1.808	0.94
None	2	3%	61	95%	1	2%	64	100%			
<2 hours	14	6%	215	92%	4	2%	233	100%			
2–4 hours	3	7%	37	90%	1	2%	41	100%			
≥4 hours	2	8%	24	92%	0	0%	26	100%			
Twitter									6	3.637	0.73
None	4	5%	76	94%	1	1%	81	100%			
<2 hours	15	7%	195	91%	4	2%	214	100%			
2–4 hours	1	2%	43	98%	0	0%	44	100%			
≥4 hours	1	4%	23	92%	1	4%	25	100%			
Facebook									6	1.72	0.94
None	19	6%	300	92%	6	2%	325	100%			
<2 hours	2	8%	24	92%	0	0%	26	100%			
2–4 hours	0	0%	6	100%	0	0%	6	100%			
≥4 hours	0	0%	7	100%	0	0%	7	100%			
YouTube									6	6.896	0.33
None	1	3%	34	97%	0	0%	35	100%			
<2 hours	15	7%	193	91%	5	2%	213	100%			
2–4 hours	5	8%	60	91%	1	2%	66	100%			
≥4 hours	0	0%	50	100%	0	0%	50	100%			

**Table 4:** Association between the desire to undergo cosmetic procedures and daily hours spent on different social media platforms *Statistically significant df: degrees of freedom

	Yes		No		Maybe		Total				
	N	%	N	%	N	%	N	%	df	Chi-squared	P-value
WhatsApp									6	12.61	0.05*
None	0	0%	0	0%	1	100%	1	100%			
<2 hours	26	11%	176	75%	33	14%	235	100%			
2–4 hours	7	9%	53	69%	17	22%	77	100%			
≥4 hours	10	20%	36	71%	5	10%	51	100%			
Snapchat									6	6.631	0.36
None	2	7%	22	81%	3	11%	27	100%			
<2 hours	16	10%	120	78%	18	12%	154	100%			
2–4 hours	16	15%	71	66%	20	19%	107	100%			
≥4 hours	9	12%	52	68%	15	20%	76	100%			
Instagram									6	5.499	0.48
None	3	5%	53	83%	8	13%	64	100%			
<2 hours	31	13%	166	71%	36	15%	233	100%			
2–4 hours	6	15%	27	66%	8	20%	41	100%			
≥4 hours	3	12%	19	73%	4	15%	26	100%			
Twitter									6	12.993	0.04*
None	5	6%	64	79%	12	15%	81	100%			
<2 hours	24	11%	157	73%	33	15%	214	100%			
2–4 hours	11	25%	24	55%	9	20%	44	100%			
≥4 hours	3	12%	20	80%	2	8%	25	100%			
Facebook									6	11.137	0.08
None	40	12%	235	72%	50	15%	325	100%			
<2 hours	1	4%	23	88%	2	8%	26	100%			
2–4 hours	0	0%	5	83%	1	17%	6	100%			
≥4 hours	2	29%	2	29%	3	43%	7	100%			
YouTube									6	3.883	0.69
None	2	6%	29	83%	4	11%	35	100%			
<2 hours	25	12%	152	71%	36	17%	213	100%			
2–4 hours	9	14%	46	70%	11	17%	66	100%			
≥4 hours	7	14%	38	76%	5	10%	50	100%			

## Discussion

Social media usage is more popular than ever, especially among young people [14]. It significantly influences the attitude toward aesthetic procedures [15]. This study aimed to assess social media's impact on people's decision-making about cosmetic procedures. Previous global studies have demonstrated that social media significantly affect people's decision-making about undergoing cosmetic procedures and may alter the level of public interest in these procedures, apart from focusing on the types of patients who seek them out [16,17]; this finding is in line with a local study among the general population of Saudi Arabia, which found that 206 out of 911 individuals were impacted by social media advertisements for plastic surgery and planned to have the procedure [18]. Surprisingly, our study results contrasted with the literature findings: most of our participants had no intentions to undergo cosmetic procedures in the future and had never undergone prior aesthetic procedures (Figure 2), despite the fact that they spent over four hours per day on social media. This outcome contradicts that of KI et al. (2019), who discussed the four-stage principle to target consumers [19]. This might be explained by the fact that Makkah society is conservative and hence not easily affected by social media.

Snapchat, YouTube, and Instagram have altered how people present themselves to the world and perceive themselves. External factors that influence the use of cosmetics include fear of age discrimination, the desire to escape racial prejudice, and force from a spouse, whether direct, subtle, or indirect. The internal factors include the desire to overcome negative emotions such as despair, humiliation, social anxiety, and the desire to appear powerful [20]. Other articles have reported certain other factors influencing people to undergo cosmetics, such as recommendations from others, social media, comparing themselves with celebrities, and psychological factors [21,22]. However, in our study, only 54 participants said they compared themselves with social media celebrities; these results are consistent with those of Alhusaini et al., who focused on Snapchat users only. In their study, more than half of the participants were not affected in terms of either actively consuming or passively watching Snapchat influencers [23].

The previous literature suggests that increased social media use will lead to a positive attitude toward cosmetic surgeries [15]. We discovered that participants who spent less time on WhatsApp and Twitter were more likely not to consider future cosmetic procedures (p<0.05, Table 4). One potential explanation is that the difference in the acceptance levels of aesthetic procedures could be related to the specific application's features and functionality. For example, WhatsApp is a messaging application enabling individuals to be in touch with close circles such as friends and family. In contrast to Snapchat, Instagram and YouTube users encounter a wider zone of social media impact because of the ability to explore and follow people outside their circle that these applications enable [15].

The popularity of cosmetic procedures has significantly increased over time. The American Society for Aesthetic Plastic Surgery’s demographic data showed that the most popular surgical procedure in 2020 was liposuction, followed by breast augmentation. Botox injection was the top non-surgical procedure [24]. In Saudi Arabia, the most popular procedure in a study among 1,678 participants was found to be laser hair removal (26.2%), followed by Botox (19%) and liposuction (14.3%) [20]. However, our study found that the most reported cosmetic procedure was dermal fillers at 43%; rhinoplasty was the most desired procedure (42%).

Strengths and limitations

This is the first study to be conducted in Makkah City, Saudi Arabia, to evaluate and provide insight into the impact of social media on attitudes toward cosmetic procedures among the general population. The sample was not limited to a specific age, gender, or social platform. To avoid recall bias, the data for this study were collected from each participant's phone set, which automatically calculates the average number of daily hours of phone usage per week and determines the average number of hours spent on almost all common social media platforms.

The major limitation of this study is that we did not include TikTok in our analysis, which has gained more popularity over the years and is considered one of the most commonly used visual social media platforms. Secondly, the sample was limited to people we encountered in Makkah City only, and hence the results cannot be generalized to the entire population of Saudi Arabia.

Recommendations

Further studies need to be conducted in other cities in Saudi Arabia because the culture and traditions may vary from those of Makkah. Moreover, future research should include the TikTok platform, which has become one of the most popular visual social media platforms. It is also important to note that this study only reported social media usage on mobile phones, and did not consider other devices that could potentially influence the results. For instance, users may spend more time on YouTube when using a larger screen device like a laptop, iPad, or desktop computer compared to a mobile phone. Future research should consider examining social media usage across multiple devices to provide a more comprehensive understanding of how it influences people's attitudes toward cosmetic procedures.

## Conclusions

Our findings indicate that even though nearly half of the participants use Snapchat for more than three hours, there is no significant association between hours spent on social media and the desire to undergo cosmetic procedures. Rhinoplasty is the most desired procedure (43%). This might be explained by the fact that Makkah society is conservative and not easily affected by social media, despite the increasing impact of social media influencers and celebrities. This study concludes that social media platforms do not impact the general population's decisions to undergo cosmetic procedures in Makkah City, Saudi Arabia.
